# Finerenone Across the Cardiovascular–Kidney–Metabolic Continuum: From Mechanistic Rationale to Clinical Positioning—A Narrative Review

**DOI:** 10.3390/jcm15093486

**Published:** 2026-05-02

**Authors:** Jacek Kubica, Aldona Kubica, Jakub Ratajczak, Robert Gajda, Łukasz Szarpak, Eliano P. Navarese

**Affiliations:** 1 Collegium Medicum, Nicolaus Copernicus University, 85-094 Bydgoszcz, Poland; jkubica@cm.umk.pl (J.K.); akubica@cm.umk.pl (A.K.); elianonavarese@gmail.com (E.P.N.); 2 Modern Medical Technologies Center, 87-100 Torun, Poland; gajda@gajdamed.pl; 3 Gajda-Med District Hospital, 06-102 Pultusk, Poland; 4 Institute of Medical Sciences, The John Paul II Catholic University of Lublin, 20-950 Lublin, Poland; lukaszszarpak@gmail.com; 5 Department of Life and Health Sciences, Link Campus University, 00165 Rome, Italy

**Keywords:** finerenone, cardiovascular–kidney–metabolic syndrome, mineralocorticoid receptor, heart failure, chronic kidney disease, diabetes mellitus

## Abstract

The cardiovascular–kidney–metabolic (CKM) syndrome has emerged as an integrated framework linking obesity, type 2 diabetes, chronic kidney disease (CKD), and heart failure with preserved or mildly reduced ejection fraction through shared mechanisms including inflammation, oxidative stress, endothelial dysfunction, and fibrosis. Persistent mineralocorticoid receptor overactivation plays a central role in this continuum, contributing to progressive cardiac and renal injury despite optimized renin–angiotensin system blockade. Finerenone, a selective non-steroidal mineralocorticoid receptor antagonist, has therefore gained increasing attention as a targeted strategy to reduce residual cardiorenal risk. This narrative review summarizes the mechanistic rationale and clinical evidence supporting finerenone across the CKM spectrum. Experimental data indicate that finerenone attenuates inflammation, fibrosis, myocardial hypertrophy, and adverse remodeling, while proteomic and translational analyses suggest biological complementarity with sodium–glucose cotransporter 2 inhibitors. Clinically, pivotal randomized trials have demonstrated that finerenone reduces kidney disease progression and major cardiovascular events in patients with CKD and type 2 diabetes, while the FINEARTS-HF trial extended these benefits to patients with heart failure with mildly reduced or preserved ejection fraction by reducing worsening heart failure events. Additional subgroup, pooled, and meta-analytic data reinforce the consistency of these effects across a broad range of cardiorenal phenotypes. Taken together, current evidence positions finerenone as an important component of contemporary CKM management, particularly in patients with diabetic CKD and selected heart failure phenotypes. Its principal value lies in targeting residual inflammatory and fibrotic risk beyond conventional hemodynamic and metabolic control. Future progress will depend on earlier phenotype recognition, improved implementation and adherence, and wider adoption of pathway-oriented combination therapy across the cardiorenal continuum.

## 1. Introduction

The concept of cardiovascular–kidney–metabolic (CKM) syndrome provides an integrated framework linking obesity, type 2 diabetes (T2D), hypertension, metabolic dysfunction–associated steatotic liver disease, chronic kidney disease (CKD), and heart failure with preserved ejection fraction (HFpEF) through shared pathophysiological mechanisms [1,2]. Within this continuum, the coexistence of metabolic, renal, and cardiovascular abnormalities accelerates vascular dysfunction, myocardial remodeling, albuminuria, and progression to overt heart failure and atherosclerotic cardiovascular disease [1,2].

A central mechanistic axis in CKM syndrome is persistent overactivation of the mineralocorticoid receptor (MR). Beyond its classical role in sodium and volume homeostasis, inappropriate MR signaling promotes inflammation, oxidative stress, endothelial dysfunction, and fibrosis in both the heart and kidneys [2,3,4]. Although blockade of the renin–angiotensin system (RAS) with angiotensin-converting enzyme (ACE) inhibitors or angiotensin receptor blockers has long been the therapeutic cornerstone, substantial residual cardiorenal risk persists despite optimized treatment. In addition, chronic RAS inhibition may be accompanied by “aldosterone breakthrough,” sustaining MR activation and contributing to progressive organ injury [3,4,5].

The therapeutic landscape has changed considerably with the advent of sodium–glucose cotransporter 2 (SGLT2) inhibitors and glucagon-like peptide-1 receptor agonists, which provide benefits extending beyond glucose lowering [6,7,8]. Within this evolving framework, non-steroidal MR antagonism has emerged as a complementary strategy aimed at residual cardiorenal risk driven by inflammatory and fibrotic pathways. Steroidal MR antagonists remain a cornerstone of therapy in heart failure with reduced ejection fraction (HFrEF), but their use in routine practice is often limited by hyperkalemia, worsening renal function, underdosing, and discontinuation [9,10]. Moreover, their role in HFpEF has remained uncertain, as exemplified by the limitations of TOPCAT [11].

These challenges have stimulated the development of more selective, non-steroidal mineralocorticoid receptor (MR) antagonists, among which finerenone is the most clinically advanced. From a clinical standpoint, the key unmet need is no longer whether cardiorenal risk can be reduced, but how to address the substantial residual risk that persists despite contemporary standard-of-care therapy. In everyday practice, this includes patients who remain albuminuric despite RAS blockade, continue to accumulate structural cardiac and renal injury despite apparently adequate blood pressure or glycemic control, and often do not fit neatly within a single specialty pathway.

In this context, finerenone should be viewed not merely as an additional therapy, but as a targeted strategy aimed at mitigating persistent non-hemodynamic drivers of disease progression, particularly inflammation and fibrosis across the cardiovascular–kidney–metabolic continuum.

Accordingly, the aim of this narrative review is to provide an integrated overview of the mechanistic rationale and clinical evidence supporting finerenone within this continuum, with particular emphasis on diabetic chronic kidney disease and HFmrEF/HFpEF, where the current evidence base is most robust.

## 2. Methods

This narrative review was conducted to provide an integrated overview of the mechanistic rationale and clinical evidence supporting the use of finerenone across the cardiovascular–kidney–metabolic continuum.

A targeted literature search was performed using PubMed/MEDLINE to identify relevant studies published up to 15 March 2026. The search focused on key terms including “finerenone”, “mineralocorticoid receptor antagonists”, “chronic kidney disease”, “type 2 diabetes”, “heart failure”, “HFpEF”, “HFmrEF”, and “cardiorenal syndrome”. Additional references were identified through manual review of bibliographies of selected articles and relevant review papers.

Priority was given to large randomized controlled trials, prespecified analyses, pooled analyses, and meta-analyses evaluating clinical outcomes associated with finerenone, particularly in populations with CKD and T2D and in HFmrEF/HFpEF. Mechanistic and translational studies were included to provide biological context where relevant.

Given the narrative nature of the review, no formal systematic selection process or quantitative evidence grading was applied. Instead, studies were selected based on their clinical relevance, methodological quality, and contribution to understanding the role of finerenone within the cardiovascular–kidney–metabolic framework.

Quality assessment of the present review by the SANRA (Scale for the Assessment of Narrative Review Articles) has been provided in the Supplementary Materials (Table S1).

## 3. From Pathophysiology to Treatment

Finerenone is a highly selective non-steroidal MR antagonist with pharmacological properties distinct from spironolactone and eplerenone, including a short plasma half-life, absence of active metabolites, and balanced distribution between cardiac and renal tissues [2,9]. These features may contribute to a more favorable tolerability profile while preserving potent anti-inflammatory and anti-fibrotic effects.

In large, randomized trials involving patients with CKD and T2D, finerenone reduced kidney disease progression and major cardiovascular events when added to optimized standard therapy [2,12]. These findings support a clinically important concept: even under appropriate RAS inhibition, substantial residual cardiorenal risk remains, and selective MR antagonism provides a means of targeting that unmet need. Importantly, the therapeutic implications of MR modulation may extend beyond advanced CKD. Increasing evidence suggests that earlier intervention along the CKM trajectory—before irreversible nephron loss or overt HFpEF—may attenuate maladaptive remodeling in both the kidney and myocardium [2].

Experimental studies provide mechanistic support for this concept. In a murine model mimicking early-stage HFpEF driven by combined metabolic and hypertensive stress, Morikawa et al. showed that finerenone enhanced glucocorticoid receptor signaling, with upregulation of cardioprotective target genes such as *Foxo3* and *Fkbp5* [13]. These findings suggest that finerenone may restore physiological balance between MR and glucocorticoid receptor signaling in cardiomyocytes, thereby mitigating hypertrophy and mitochondrial dysfunction. In parallel, preclinical studies relevant to HFpEF have shown that finerenone attenuates myocardial inflammation and fibrosis, reduces hypertrophy, lowers natriuretic peptide levels, and improves functional parameters consistent with early systolic and diastolic benefit; in several models, its effects on cardiac remodeling appeared more pronounced than those of eplerenone at equivalent natriuretic doses [5].

**Figure 1:**
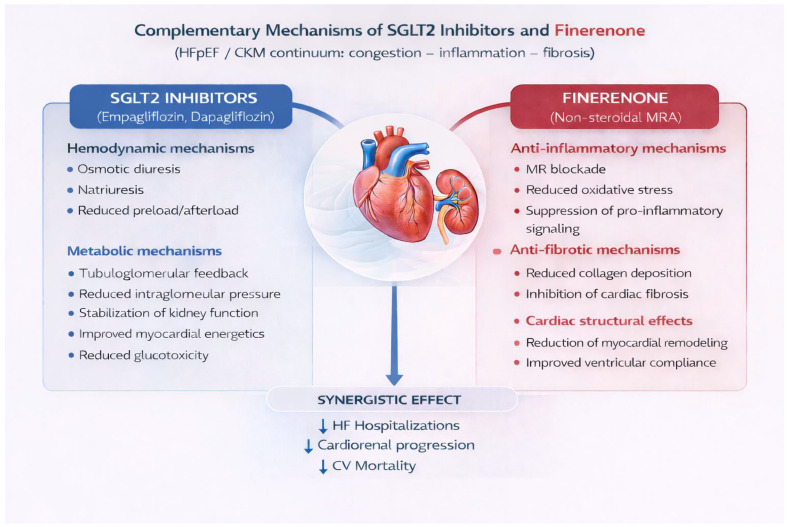
Complementary Mechanisms of SGLT2 Inhibitors and Finerenone. The conceptual design and scientific content were created by the authors, and AI-assisted tools were used solely for graphical rendering.

Additional molecular evidence indicates that finerenone and SGLT2 inhibitors target distinct but complementary biological pathways. Proteomic analyses comparing dapagliflozin and finerenone suggest that dapagliflozin predominantly influences metabolic, tubular, and hemodynamic pathways, whereas finerenone more specifically modulates inflammatory, fibrotic, and extracellular matrix signaling [14]. The limited overlap between these proteomic signatures supports the rationale for multidrug, pathway-oriented cardiorenal protection [14,15,16] (Figure 1).

Taken together, these data position finerenone as a mechanistically coherent intervention within the CKM framework, targeting the inflammatory–fibrotic axis that links metabolic dysfunction, subclinical renal injury, myocardial hypertrophy, and HFpEF.

## 4. Clinical Evidence

### 4.1. Pivotal Randomized Trials

The clinical evidence supporting finerenone is derived from several large, randomized trials. For clarity, the key design features, efficacy outcomes, and safety profiles of the pivotal finerenone trials are summarized in Table 1.

The FIDELIO-DKD trial evaluated finerenone in 5734 patients with CKD and T2D receiving an ACE inhibitor or angiotensin receptor blocker at the maximum tolerated labeled dose [12]. Finerenone significantly reduced the primary composite kidney outcome—kidney failure, sustained ≥40% decline in estimated glomerular filtration rate (eGFR), or renal death—compared with placebo (17.8% vs. 21.1%; HR 0.82, 95% CI 0.73–0.93; *p* = 0.001). The benefit was consistent across HbA1c strata and irrespective of insulin use [25]. Finerenone also reduced the key secondary cardiovascular composite outcome of cardiovascular death, nonfatal myocardial infarction, nonfatal stroke, or hospitalization for heart failure (13.0% vs. 14.8%; HR 0.86, 95% CI 0.75–0.99; *p* = 0.03), with directionally favorable effects on cardiovascular death, myocardial infarction, and hospitalization for heart failure [26]. Additional analyses showed reduced hospitalization for heart failure even in patients without overt heart failure at baseline and preserved benefit irrespective of concomitant GLP-1 receptor agonist therapy [27,28]. Finerenone also induced an early and sustained reduction in albuminuria, with an approximately 31% greater reduction in urinary albumin-to-creatinine ratio at month 4 versus placebo [12]. Hyperkalemia was more frequent with finerenone (18.3% vs. 9.0%), although overall and serious adverse event rates were similar between groups [12]. A subsequent analysis also demonstrated a reduction in new-onset atrial fibrillation, suggesting anti-remodeling effects beyond kidney protection [29].

The FIGARO-DKD trial extended these observations to a broader CKD population, enrolling 7437 patients with T2D and earlier-stage CKD or less advanced albuminuria [17]. Over a median follow-up of 3.4 years, finerenone significantly reduced the primary cardiovascular outcome—cardiovascular death, nonfatal myocardial infarction, nonfatal stroke, or hospitalization for heart failure—compared with placebo (12.4% vs. 14.2%; HR 0.87, 95% CI 0.76–0.98; *p* = 0.03). This effect was driven mainly by fewer hospitalizations for heart failure (HR 0.71, 95% CI 0.56–0.90) [17]. In prespecified analyses, finerenone reduced the risk of incident heart failure by 32% among patients without prior heart failure (HR 0.68, 95% CI 0.50–0.93), corresponding to an absolute risk reduction of 1.1% at 48 months [30].

The FIDELITY prespecified pooled analysis combined individual patient data from FIDELIO-DKD and FIGARO-DKD, including more than 13,000 patients with CKD and T2D receiving optimized RAS blockade [18]. Finerenone reduced both kidney disease progression and major cardiovascular events, confirming its role in comprehensive cardiorenal risk reduction. Importantly, treatment effects were consistent irrespective of background SGLT2 inhibitor use, with no significant interaction between therapies and numerical trends suggesting additive benefit [19]. Hyperkalemia remained more frequent with finerenone, but discontinuation due to hyperkalemia was uncommon and appeared numerically lower among patients receiving concomitant SGLT2 inhibitors [19]. Additional FIDELITY analyses confirmed consistent benefits across BMI categories and across strata of eGFR and albuminuria, including reductions in first and recurrent heart failure hospitalization [31,32].

The FINEARTS-HF trial extended finerenone into symptomatic heart failure with mildly reduced ejection fraction (HFmrEF) or HFpEF. In 6001 patients treated on top of guideline-directed therapy, finerenone reduced the primary composite of total worsening heart failure events and cardiovascular death by 16% versus placebo (rate ratio 0.84, 95% CI 0.74–0.95; *p* = 0.007), driven mainly by fewer worsening heart failure events [20]. The effect was consistent across age, sex, kidney function, LVEF strata, geographic region, and background heart failure therapies [20,21]. Hyperkalemia occurred more often with finerenone, but severe events were uncommon, potassium-related hospitalizations were rare, and no deaths were attributed to potassium disturbances [21]. Time-updated analyses showed that finerenone retained clinical benefit even after potassium exceeded 5.5 mmol/L, suggesting that modest potassium elevations should not automatically prompt treatment discontinuation [21]. Although an early, modest decline in eGFR was observed, the subsequent rate of decline was slower with finerenone, consistent with an overall favorable cardiorenal profile [22,23]. Additional prespecified analyses demonstrated consistent benefits irrespective of adiposity, NYHA class, prior LVEF trajectory, recent worsening heart failure, glycemic status, and baseline SGLT2 inhibitor use [33,34,35,36,37,38]. Modeling analyses further suggested that finerenone may translate relative risk reductions into clinically meaningful gains in event-free survival, particularly when initiated earlier in life, although such projections necessarily assume durable treatment effect and trial-level adherence [39].

Collectively, FIDELIO-DKD, FIGARO-DKD, FIDELITY, and FINEARTS-HF position finerenone as an important component of contemporary cardiorenal therapy. The importance of FINEARTS-HF is therefore broader than the primary endpoint alone. It provides the first robust confirmation that selective non-steroidal MR antagonism can translate biologically plausible anti-fibrotic and anti-inflammatory effects into clinically meaningful benefit in patients with HFmrEF/HFpEF, a population historically characterized by therapeutic heterogeneity and modest treatment responsiveness. In CKD associated with T2D, finerenone reduces kidney disease progression and improves cardiovascular outcomes on top of optimized RAS inhibition. In HFmrEF/HFpEF, it lowers the risk of worsening heart failure events with a manageable safety profile under appropriate monitoring.

### 4.2. Limitations of Pivotal Trials and Implications for Clinical Practice

While the pivotal trials of finerenone provide robust evidence supporting its cardiovascular and renal benefits, several limitations should be acknowledged when translating these findings into routine clinical practice. First, the study populations were highly selected, typically including patients with type 2 diabetes and chronic kidney disease under stable background therapy, which may limit generalizability to broader and more heterogeneous populations, including those with non-diabetic CKD or more advanced heart failure phenotypes. Second, patients at highest risk of hyperkalemia or with more severe renal impairment were often excluded or underrepresented, potentially leading to an underestimation of safety challenges in real-world settings. Third, follow-up durations, while sufficient to demonstrate efficacy, may not fully capture long-term outcomes and safety signals. Finally, the controlled environment of randomized clinical trials, characterized by close monitoring and high adherence, differs substantially from routine clinical practice, where variability in patient behavior, comorbidities, and healthcare system factors may influence both effectiveness and safety. These considerations underscore the importance of careful patient selection, structured monitoring, and the complementary role of real-world evidence in refining the clinical use of finerenone.

### 4.3. Complementary and Real-World Evidence

Before the phase III cardiorenal program, ARTS-HF compared finerenone with eplerenone in patients with worsening HFrEF and concomitant T2D and/or CKD [24]. Finerenone achieved similar reductions in NT-proBNP at day 90 and showed numerically lower rates of cardiovascular death, hospitalization for heart failure, or urgent heart failure visits in selected dose groups, with low and comparable rates of hyperkalemia-related discontinuation [24]. These findings provided an early clinical rationale for subsequent outcome trials.

Real-world evidence has broadly supported these observations. In a large multicenter cohort, non-steroidal MR antagonists—predominantly finerenone—were associated with lower rates of heart failure hospitalization or cardiovascular death than steroidal MR antagonists (HR 0.79, 95% CI 0.70–0.90; *p* < 0.001), with fewer serious hyperkalemia events and fewer hormone-related adverse effects [40]. Pharmacovigilance analyses likewise suggest a class effect for hyperkalemia and renal dysfunction, but fewer off-target adverse events with finerenone than with spironolactone, consistent with its higher receptor selectivity [41].

Ongoing trials are now testing whether complementary mechanisms can be translated into greater clinical benefit. In the CONFIDENCE trial, patients with CKD and T2D receiving optimized RAS blockade have been randomized to finerenone plus empagliflozin, finerenone alone, or empagliflozin alone, providing a direct test of whether early combination therapy yields superior albuminuria reduction and cardiorenal protection [42].

At the same time, real-world comparisons between steroidal and non-steroidal MR antagonists should be interpreted with appropriate caution, as treatment selection, monitoring intensity, and baseline risk may differ substantially across cohorts. Nevertheless, the overall direction of evidence is clinically reassuring and aligns with the pharmacological rationale for improved tolerability with finerenone.

### 4.4. Meta-Analyses

Meta-analytic evidence supports and contextualizes the individual trial findings. Zhang et al. analyzed nine randomized trials including 33,128 participants and showed that MR antagonists reduce cardiovascular events, heart failure hospitalization, and mortality across the heart failure spectrum, with the largest effects in HFrEF and more modest but still clinically relevant benefits in HFmrEF/HFpEF [4]. Notably, subgroup analyses suggested a stronger signal for finerenone than for spironolactone in higher-ejection-fraction populations, particularly for cardiovascular composite outcomes [4].

A complementary meta-analysis by Wu et al., based on six randomized trials including 20,699 patients, confirmed that MR antagonists reduce the composite of heart failure hospitalization or cardiovascular death, as well as heart failure hospitalization, cardiovascular mortality, all-cause mortality, and sudden cardiac death [43]. As expected, hyperkalemia, hypotension, and worsening renal function occurred more frequently, whereas hypokalemia was reduced [43].

Beyond heart failure populations, Chen et al. analyzed seven randomized trials in diabetic kidney disease and found that finerenone significantly reduced albuminuria, kidney failure, and end-stage kidney disease, while maintaining overall adverse event rates comparable to placebo apart from a higher incidence of hyperkalemia [44]. Similarly, Peng et al. showed that finerenone reduced heart failure occurrence or hospitalization across populations with HF, CKD, and T2D, although mortality reductions were not statistically significant [45]. A broader conventional and network meta-analysis by Shokravi et al. integrated 33 trials and showed that finerenone has the clearest and most consistent benefits in diabetic CKD, with emerging but less extensive evidence in LVEF ≥ 40% heart failure populations [15].

Overall, these analyses reinforce that finerenone provides clinically meaningful renal and cardiovascular protection, particularly in CKD associated with T2D, and that its effects are biologically complementary to those of SGLT2 inhibitors and other components of modern cardiorenal therapy [1,2,15]. Importantly, meta-analytic consistency should not be interpreted as equivalence across clinical settings. The strongest and most mature evidence remains in CKD associated with T2D, whereas the expanding HFmrEF/HFpEF signal, although compelling, is still less extensive and should be integrated with phenotype-specific clinical judgment.

### 4.5. Practical Considerations for Finerenone Initiation and Monitoring

The clinical use of finerenone requires careful patient selection and structured monitoring to maximize benefit while minimizing the risk of hyperkalemia and renal function changes. A structured clinical approach to finerenone initiation and monitoring is summarized in Table 2.

In routine practice, finerenone is most commonly considered in patients with CKD associated with type 2 diabetes who remain albuminuric despite optimized renin–angiotensin system inhibition, as well as in selected patients with HFmrEF/HFpEF and evidence of cardiorenal involvement.

Baseline assessment of kidney function and serum potassium is essential prior to treatment initiation, followed by early reassessment and periodic monitoring thereafter. Dose selection is typically guided by baseline renal function, with subsequent titration according to tolerance and laboratory parameters. Mild increases in potassium are relatively frequent but are usually manageable with monitoring and appropriate adjustment of concomitant therapies rather than treatment discontinuation.

Within contemporary cardiorenal therapy, finerenone should be viewed as a complementary, pathway-oriented intervention, typically added on top of optimized RAS inhibition and, when appropriate, in combination with SGLT2 inhibitors.

## 5. Discussion

### 5.1. Why Finerenone?

The contemporary understanding of CKM syndrome has shifted the therapeutic focus away from isolated organ-based management toward integrated, mechanism-oriented care. This change is particularly relevant in phenotypes such as diabetic CKD, HFmrEF, and HFpEF, where inflammation, oxidative stress, endothelial dysfunction, and fibrosis play a central role in disease progression [1,2,6]. In these settings, progression is often not driven solely by conventional hemodynamic or metabolic abnormalities, but also by persistent maladaptive signaling pathways that continue to promote structural injury despite apparently appropriate control of blood pressure, glycemia, or volume status.

Within this framework, MR overactivation has emerged as a particularly important pathophysiological mechanism. Beyond its classical effects on sodium handling and volume regulation, sustained MR signaling contributes directly to inflammation, oxidative stress, endothelial dysfunction, myocardial and renal fibrosis, and adverse remodeling [2,3,4,5]. This is clinically important because it helps explain why substantial cardiorenal risk persists even in patients receiving optimized renin–angiotensin system (RAS) blockade and other components of guideline-directed therapy. In other words, contemporary treatment may control major upstream drivers of disease while leaving downstream inflammatory–fibrotic mechanisms insufficiently addressed.

Finerenone is therefore of interest not merely because it belongs to the mineralocorticoid receptor antagonist class, but because it offers a pharmacologically distinct, non-steroidal approach to MR blockade. Compared with steroidal agents, finerenone has greater receptor selectivity, no active metabolites, and a more balanced distribution between cardiac and renal tissues, features that may contribute to a more favorable tolerability profile while preserving anti-inflammatory and anti-fibrotic activity [2,9]. This distinction is clinically relevant, as the underuse of steroidal MR antagonists in routine practice has often been driven not by lack of efficacy, but by concerns related to hyperkalemia, renal function deterioration, endocrine adverse effects, underdosing, and premature discontinuation [9,10]. In that sense, finerenone represents not simply a reformulation of an established concept, but an attempt to improve the applicability of MR-targeted therapy across a broader cardiorenal population.

The rationale for finerenone has become even stronger in the era of SGLT2 inhibitors, which have reshaped the treatment of heart failure and diabetic kidney disease. SGLT2 inhibitors were the first class to demonstrate consistent reductions in the composite of cardiovascular death or heart failure hospitalization across the ejection fraction spectrum, with benefits largely driven by fewer heart failure events [6]. These results established SGLT2 inhibitors as a foundational component of cardiorenal therapy. However, the success of SGLT2 inhibition has also clarified an important clinical reality: even highly effective therapies do not abolish residual risk. Many patients continue to progress, remain albuminuric, accumulate myocardial and renal structural damage, or experience recurrent heart failure events despite treatment with agents that are now considered standard of care.

It is in this context that finerenone appears particularly relevant. Mechanistically, it seems well aligned with the dominant biology of CKM syndrome and higher-ejection-fraction heart failure phenotypes, where inflammation, fibrosis, and cellular remodeling are more central than marked volume overload or severe systolic impairment. Experimental studies further support this concept. In preclinical models, finerenone attenuates myocardial inflammation, fibrosis, hypertrophy, and adverse remodeling, while improving parameters consistent with early systolic and diastolic benefit [5,13]. The study by Morikawa et al. adds a further layer of mechanistic plausibility by suggesting that finerenone may restore balance between MR and glucocorticoid receptor signaling in cardiomyocytes, with downstream effects on hypertrophy and mitochondrial homeostasis [13]. These observations are especially relevant in HFpEF and related cardiometabolic phenotypes, where therapeutic progress has historically been hindered by biological heterogeneity and the absence of clearly targetable dominant pathways.

Another important element is the apparent biological complementarity between finerenone and SGLT2 inhibitors. Proteomic and translational analyses suggest that these therapies do not simply duplicate one another’s effects [14]. Rather, SGLT2 inhibitors appear to act predominantly through hemodynamic, tubular, natriuretic, and metabolic mechanisms, whereas finerenone more directly influences inflammatory, fibrotic, and extracellular matrix pathways [14,15,16]. This distinction has important conceptual implications. It suggests that finerenone should not be viewed as competing with established therapies for the same therapeutic niche, but rather as filling a mechanistic gap within an increasingly multidimensional treatment strategy. Accordingly, the justification for finerenone is strongest not in the abstract question of whether MR blockade is biologically interesting, but in the practical reality that a substantial proportion of patients remain exposed to persistent cardiorenal injury despite otherwise appropriate treatment.

### 5.2. For Whom?

The clearest answer to this question comes from the populations studied in the major randomized trials. The most robust and mature evidence supports the use of finerenone in patients with CKD associated with T2D, particularly those who remain albuminuric despite optimized RAS inhibition [12,17,18]. Across FIDELIO-DKD, FIGARO-DKD, and the pooled FIDELITY analysis, finerenone consistently reduced kidney disease progression and major cardiovascular outcomes, with particularly relevant effects on hospitalization for heart failure and progression of albuminuric kidney disease [12,17,18,19,30,31,32]. These data define a clinically important phenotype: the patient with diabetic CKD who remains at high residual risk despite background therapy that would traditionally be considered appropriate.

This phenotype is highly relevant in routine practice. Such patients often have partial but incomplete control of conventional risk factors, yet continue to demonstrate persistent albuminuria, progressive eGFR decline, subclinical structural cardiac abnormalities, or a growing burden of cardiovascular events. From a practical standpoint, finerenone is particularly attractive in this population because it targets processes not fully addressed by glucose lowering, blood pressure control, or RAS inhibition alone. The benefits observed in the pivotal trials therefore appear to reflect more than incremental pharmacological layering; rather, they support the concept that selective MR antagonism can modify clinically meaningful pathways of ongoing organ injury in patients who remain vulnerable despite otherwise contemporary care.

The second major group for whom finerenone appears relevant comprises patients with symptomatic HFmrEF/HFpEF, particularly those with overlapping cardiometabolic and renal features. The FINEARTS-HF trial extended finerenone into this setting by demonstrating a significant reduction in the composite of total worsening heart failure events and cardiovascular death, an effect driven mainly by fewer worsening heart failure events [20]. This finding is important not only because it adds another therapeutic option in a field historically characterized by limited responsiveness, but also because it provides clinical confirmation that selective non-steroidal MR antagonism can translate biologically plausible anti-inflammatory and anti-fibrotic effects into measurable benefit in patients with LVEF ≥ 40%.

This population deserves particular attention. HFmrEF/HFpEF is not a single disease entity, but a heterogeneous syndrome encompassing varying combinations of obesity, hypertension, diabetes, CKD, atrial dysfunction, vascular stiffness, microvascular inflammation, and extracardiac congestion. In many patients, especially those with cardiometabolic or cardiorenal profiles, inflammation and fibrosis seem to be central disease drivers. It is therefore plausible that finerenone may be especially well suited to phenotypes characterized by overlapping renal dysfunction, metabolic stress, persistent congestion risk, and structural remodeling. This interpretation is supported by prespecified analyses from FINEARTS-HF showing consistent effects across age, sex, kidney function, adiposity, glycemic status, LVEF strata, prior worsening heart failure, and background SGLT2 inhibitor use [21,22,23,33,34,35,36,37,38,39]. Although such subgroup analyses should always be interpreted cautiously, their overall consistency strengthens the impression that finerenone may have broad applicability within selected higher-ejection-fraction heart failure phenotypes.

At the same time, the boundaries of the evidence base should be clearly acknowledged. The strongest support remains in CKD associated with T2D and in HFmrEF/HFpEF. Outside these groups, the data are less well known. Extrapolation to non-diabetic CKD, advanced or unstable heart failure phenotypes, or other broader CKM populations should therefore be cautious and clinically reasoned rather than automatic. This point is particularly important given the increasingly expansive conceptual language surrounding CKM syndrome. While the syndrome itself spans a wide biological and clinical continuum, the trial-based evidence for finerenone does not yet cover that continuum uniformly. Thus, although finerenone is highly relevant within the CKM framework, its current evidence-based positioning remains concentrated in specific, better-defined phenotypes.

A further practical consideration is that trial populations may not fully reflect the complexity of real-world patients. Individuals at highest risk of hyperkalemia, with more severe renal dysfunction, multimorbidity, polypharmacy, or healthcare fragmentation may be underrepresented in randomized trials. As a result, the ideal candidate for finerenone in everyday practice is not simply anyone meeting broad disease labels, but rather a patient in whom the expected cardiorenal benefit is meaningful and the monitoring framework is realistic. In this respect, “for whom” should be answered not only by diagnosis, but by phenotype: persistent albuminuria despite RAS inhibition, diabetic CKD with ongoing progression risk, HFmrEF/HFpEF with cardiorenal-metabolic overlap, and a clinical setting in which structured potassium and renal surveillance can be ensured.

### 5.3. How Does Finerenone Fit into Clinical Practice?

From a practical standpoint, finerenone should be positioned as a complementary component of contemporary cardiorenal therapy rather than as a replacement for established treatments. Its role is best understood within a layered, pathway-oriented treatment model in which therapies are selected not only on the basis of diagnosis, but also according to the dominant mechanisms of residual risk. In this framework, RAS inhibition remains foundational, SGLT2 inhibitors provide broad cardiorenal benefit, and finerenone offers an additional strategy for targeting persistent MR-driven inflammation and fibrosis. This is particularly relevant in patients who continue to demonstrate albuminuria, progressive renal dysfunction, recurrent heart failure events, or broader evidence of structural organ injury despite otherwise appropriate care.

This positioning has several important implications. First, finerenone should be considered early enough to influence disease trajectories before irreversible remodeling becomes advanced. The clinical value of cardiorenal therapies is often greatest when introduced before the accumulation of extensive nephron loss, repeated decompensation, or fixed myocardial stiffness. While the pivotal trials enrolled patients with established disease, their mechanistic rationale strongly supports earlier recognition of patients with high-risk phenotypes rather than delayed use after progressive deterioration. Second, finerenone appears particularly suitable for combination therapy. Mechanistic, proteomic, and exploratory clinical data suggest complementarity with SGLT2 inhibitors, reinforcing the concept that multidrug strategies addressing different biological pathways may offer more complete protection than any single therapy alone [14,15,16,19,38,42,46].

The practical barriers to implementation are therefore less about uncertainty of rationale and more about clinical confidence, workflow integration, and monitoring. Hyperkalemia remains the most frequently cited concern, and appropriately so. Across trials, potassium elevations were more common with finerenone than with placebo, although severe events were infrequent, hospitalizations were rare, and treatment discontinuation rates remained relatively low under structured monitoring [12,17,18,20,21]. Importantly, this pattern should be interpreted carefully. The signal does not suggest that finerenone is intrinsically difficult to use, but rather that its safe implementation depends on routine principles of good clinical practice: baseline assessment of eGFR and serum potassium, dose selection according to renal function, early laboratory reassessment after initiation or dose change, and periodic follow-up thereafter. Mild potassium increases are often manageable through closer observation and adjustment of concomitant therapies rather than immediate discontinuation.

This is an important practical message because fear of hyperkalemia has historically contributed to the underuse of MR antagonism in patients most likely to benefit. Finerenone may help address some of these concerns because of its selectivity and favorable tolerability profile relative to steroidal agents, but it does not eliminate the need for disciplined monitoring. Rather, it shifts the clinical question from “Is this drug too risky?” to “Can this therapy be implemented appropriately?” In most eligible patients, the answer is yes, provided that basic monitoring infrastructure is in place. From that perspective, potassium management should be framed as a manageable component of therapy rather than as a reason for routine therapeutic avoidance.

Another important issue is the difference between efficacy and implementation. Randomized trials are conducted in structured environments with protocolized follow-up, high adherence, and close biochemical surveillance. Routine practice is more variable. Patients may receive fragmented care across specialties, laboratory checks may be delayed, and adherence may be inconsistent [47,48,49,50,51]. This matters because the real-world effectiveness of finerenone, as with any cardiorenal therapy, depends not only on biological efficacy but also on the quality of implementation. In this respect, the broader lesson extends beyond finerenone itself. The future of CKM care will likely depend increasingly on pathway-based models that integrate nephrology, cardiology, diabetology, and primary care, supported by structured follow-up and clearer ownership of treatment titration and laboratory monitoring. The need is therefore not simply for another effective drug, but for care systems capable of using effective drugs well.

The question of how finerenone fits into practice also includes how clinicians should interpret emerging evidence beyond the pivotal trials. ARTS-HF provided an early signal that finerenone could achieve clinically relevant neurohormonal effects with acceptable tolerability in patients with worsening HFrEF and concomitant T2D and/or CKD [24]. Real-world and pharmacovigilance data have further suggested lower rates of certain off-target adverse effects and a reassuring overall tolerability profile relative to steroidal MR antagonists, although such comparisons must be interpreted cautiously because of residual confounding and differences in treatment selection [40,41]. Meanwhile, ongoing studies such as CONFIDENCE are exploring whether simultaneous initiation with SGLT2 inhibitors may enhance cardiorenal protection [42]. Taken together, these data support a dynamic therapeutic position for finerenone: no longer an emerging option of purely theoretical promise, but an increasingly established component of integrated cardiorenal care whose precise use will continue to evolve as combination strategies become better defined.

Finally, finerenone should be interpreted within the broader transition from organ-specific to continuum-based care. Historically, patients with diabetic CKD, HFmrEF, or HFpEF were often managed within separate specialty silos, with treatment decisions framed narrowly around glucose lowering, blood pressure control, or volume management. The CKM paradigm has challenged this fragmentation by emphasizing shared biology and overlapping trajectories of renal and cardiovascular injury. Finerenone fits particularly well within this newer model because its value lies precisely in addressing mechanisms that cut across traditional specialty boundaries. It is not primarily a glucose-lowering drug, a diuretic, or a conventional hemodynamic therapy. Instead, it is a targeted intervention for ongoing inflammatory–fibrotic injury in patients whose disease burden spans the cardiovascular, renal, and metabolic domains.

Overall, the available evidence suggests that finerenone has moved from being a mechanistically attractive therapy to one supported by a coherent and clinically meaningful body of evidence. Its principal strength lies in addressing persistent cardiorenal risk that remains despite contemporary standard-of-care therapy, especially in diabetic CKD and selected HFmrEF/HFpEF phenotypes. Its implementation in practice is feasible, but depends on phenotype recognition, thoughtful therapeutic positioning, and structured laboratory surveillance. The central challenge is therefore no longer whether finerenone has a role, but how best to identify the patients in whom its biological rationale, evidence base, and practical usability align most convincingly.

## 6. Conclusions

Finerenone represents a mechanistically distinct, non-steroidal mineralocorticoid receptor antagonist that addresses residual cardiovascular and renal risk along the cardiorenal continuum, particularly in patients with chronic kidney disease and type 2 diabetes. Evidence from pivotal randomized trials, supported by real-world data and meta-analyses, consistently demonstrates its ability to reduce the risk of kidney disease progression and major cardiovascular outcomes, with a safety profile that is manageable under appropriate monitoring.

From a clinical perspective, the integration of finerenone into contemporary cardiorenal therapy requires careful patient selection, baseline assessment of renal function and serum potassium, and structured follow-up to mitigate the risk of hyperkalemia. Rather than replacing established therapies, finerenone should be viewed as a complementary component of guideline-directed treatment, particularly in patients with persistent residual risk despite optimized standard care.

Future research should further define its role across broader patient populations, including non-diabetic CKD and heart failure phenotypes, and clarify its positioning within increasingly complex, multidimensional treatment pathways targeting the cardiovascular–kidney–metabolic axis.

## Figures and Tables

**Table 1:** Key randomized trials of finerenone across the cardiorenal spectrum.

Trial	Population	Key Inclusion	Primary Endpoint	Main Outcomes	Safety
FIDELIO-DKD [12]	CKD + T2D (n = 5734)	Albuminuric CKD; on ACEi/ARB	Kidney composite (KF, ≥40% ↓ eGFR, renal death)	↓ kidney events: HR 0.82; ↓ CV composite: HR 0.86	Hyperkalemia: 18.3% vs. 9.0%; discontinuation low
FIGARO-DKD [17]	CKD + T2D (n = 7437)	Earlier CKD/lower albuminuria	CV composite (CV death, MI, stroke, HHF)	↓ CV events: HR 0.87 (driven by ↓ HHF)	↑ hyperkalemia; low discontinuation
FIDELITY [18,19]	Pooled FIDELIO + FIGARO (>13,000)	Broad CKD + T2D spectrum	Kidney and CV composites	Consistent ↓ kidney and CV outcomes; additive with SGLT2i	↑ hyperkalemia; discontinuation uncommon
FINEARTS-HF [20,21,22,23]	HFmrEF/HFpEF (n = 6001)	Symptomatic HF, LVEF ≥ 40%	Total worsening HF events + CV death	↓ primary endpoint: RR 0.84 (mainly ↓ HF events)	↑ hyperkalemia; severe events rare
ARTS-HF [24]	HFrEF + T2D/CKD (n = 1066)	Worsening HFrEF	NT-proBNP reduction	Similar NT-proBNP vs. eplerenone; favorable clinical trends	Low hyperkalemia-related discontinuation

Abbreviations: ACEi—angiotensin-converting enzyme inhibitor; ARB—angiotensin receptor blocker; CKD—chronic kidney disease; CV—cardiovascular; eGFR—estimated glomerular filtration rate; HF—heart failure; HHF—hospitalization for heart failure; KF—kidney failure; LVEF—left ventricular ejection fraction; T2D—type 2 diabetes.

**Table 2:** Clinical checklist for finerenone initiation and monitoring.

Step	Key Considerations	Practical Notes
Patient selection	CKD with T2D and persistent albuminuria despite RAS inhibition; selected HFmrEF/HFpEF patients	Consider especially in patients with residual cardiorenal risk
Baseline assessment	eGFR and serum potassium	Avoid initiation in patients with significantly elevated potassium
Initiation	Dose guided by renal function	Lower starting dose in reduced eGFR
Early follow-up	Reassess potassium and renal function after initiation or dose change	Typically within the first weeks
Ongoing monitoring	Periodic potassium and eGFR assessment	Frequency individualized based on risk profile
Management of mild hyperkalemia	Continue treatment with closer monitoring; adjust concomitant drugs	Temporary dose reduction or interruption if needed
Therapeutic positioning	Add-on to RAS inhibition; consider combination with SGLT2 inhibitors	Mechanistically complementary approach

Abbreviations: CKD—chronic kidney disease; T2D—type 2 diabetes; HFmrEF—heart failure with mildly reduced ejection fraction; HFpEF—heart failure with preserved ejection fraction; RAS—renin–angiotensin system; eGFR—estimated glomerular filtration rate.

## Data Availability

No new data were created or analyzed in this study.

## Supplementary Materials

The following supporting information can be downloaded at: https://www.mdpi.com/article/10.3390/jcm15093486/s1, Table S1: SANRA (Scale for the Assessment of Narrative Review Articles) quality assessment of the present review.

## Abbreviations

The following abbreviations are used in this manuscript:

CKM	cardiovascular–kidney–metabolic
CKD	chronic kidney disease
T2D	type 2 diabetes
HFpEF	heart failure with preserved ejection fraction
MR	mineralocorticoid receptor
RAS	renin–angiotensin system
ACE	angiotensin-converting enzyme
SGLT2i	sodium–glucose cotransporter 2 inhibitors
HFrEF	heart failure with reduced ejection fraction
eGFR	estimated glomerular filtration rate
HFmrEF	heart failure with mildly reduced ejection fraction
