# Supplementary material for: Finerenone Across the Cardiovascular–Kidney–Metabolic Continuum: From Mechanistic Rationale to Clinical Positioning—A Narrative Review
Source: J Clin Med. 2026 May 2;15(9):3486. doi: 10.3390/jcm15093486 (PMC13163276; doi:10.3390/jcm15093486)
Supplement: Supplementary file 1 [file jcm-15-03486-s001.zip › jcm-4248296-supplementary.pdf]

Supplementary Materials

Supplementary Materials

Table S1. SANRA (Scale for the Assessment of Narrative Review Articles) quality assessment of the present review.

SANRA item	Score	Justification
1. Justification of the article's importance for the readership	2	The manuscript addresses a clinically relevant topic of finerenone within the cardiovascular–kidney–metabolic continuum.
2. Statement of concrete aims or formulation of questions	2	The aims are clearly stated, integrating mechanistic and clinical evidence.
3. Description of the literature search	1	A targeted search strategy is described, though not fully systematic, consistent with a narrative review.
4. Referencing	2	Comprehensive and up-to-date references including pivotal trials and meta-analyses.
5. Scientific reasoning	2	Coherent synthesis of mechanistic and clinical evidence.
6. Appropriate presentation of data	2	Key evidence summarized in structured tables and figures.

Total score: 11/12 (high methodological quality).
